# Testing Insecticidal Activity of Novel Chemically Synthesized siRNA against *Plutella xylostella* under Laboratory and Field Conditions

**DOI:** 10.1371/journal.pone.0062990

**Published:** 2013-05-07

**Authors:** Liang Gong, Yong Chen, Zhen Hu, Meiying Hu

**Affiliations:** Key Laboratory of Pesticide and Chemical Biology, College of Natural Resources and Environment, South China Agricultural University, Guangzhou, China; Cankiri Karatekin University, Turkey

## Abstract

**Background:**

Over the last 60 years, synthetic chemical pesticides have served as a main tactic in the field of crop protection, but their availability is now declining as a result of the development of insect resistance. Therefore, alternative pest management agents are needed. However, the demonstration of RNAi gene silencing in insects and its successful usage in disrupting the expression of vital genes opened a door to the development of a variety of novel, environmentally sound approaches for insect pest management.

**Methodology/Principal Findings:**

Six small interfering RNAs (siRNAs) were chemically synthesized and modified according to the cDNA sequence of *P. xylostella* acetylcholine esterase genes AChE1 and AChE2. All of them were formulated and used in insecticide activity screening against *P. xylostella*. Bioassay data suggested that Si-ace1_003 and Si-ace2_001 at a concentration of 3 µg cm^−2^ displayed the best insecticidal activity with 73.7% and 89.0%, mortality, respectively. Additional bioassays were used to obtain the acute lethal concentrations of LC50 and LC90 for Si-ace2_001, which were 53.66 µg/ml and 759.71 µg/ml, respectively. Quantitative Real-time PCR was used to confirm silencing and detected that the transcript levels of *P. xylostella* AChE2 (*PxAChE2*) were reduced by 5.7-fold compared to the control group. Consequently, AChE activity was also reduced by 1.7-fold. Finally, effects of the siRNAs on treated plants of *Brassica oleracea* and *Brassica alboglabra* were investigated with different siRNA doses. Our results showed that Si-ace2_001 had no negative effects on plant morphology, color and growth of vein under our experimental conditions.

**Conclusions:**

The most important finding of this study is the discovery that chemically synthesized and modified siRNA corresponding to *P. xylostella* AChE genes cause significant mortality of the insect both under laboratory and field conditions, which provides a novel strategy to control *P. xylostella* and to develop bio-pesticides based on the RNA interference technology.

## Introduction

Acetylcholinesterase (AChE, EC3.1.1.7) is located at the synapses of cholinergic neurons in the central and peripheral nervous systems in all animals. It is essential for catalyzing the hydrolysis of the neurotransmitter acetylcholine (ACh) and terminating neurotransmission [1], [2]. Based on the strategy of inhibiting AChE, both organophosphate and carbamate insecticides have been developed to control various insect species such as the diamondback moth (DBM), *Plutella xylostella* (Lepidoptera: Plutellidae) [3], [4]. This insect is the most destructive pest of cruciferous plants, especially cabbage, in many parts of the world, and the annual cost for its management has been estimated to be up to US $ 4–5 billion [5], [6]. Use of chemically synthesized insecticides remains the main control strategy for *P. xylostella* due to their easy application and cost-effectiveness. Unfortunately, continuous massive use of organophosphate and carbamate insecticides from the 1980s has brought upon serious resistance to these insecticides in *P. xylostella* [7]–[9]. Furthermore, current reports demonstrate that some *P. xylostella* populations have evolved resistance to almost every chemically synthesised insecticide class applied in the field, including organophosphates, carbamates, pyrethroids, spinosyns, avermectins, neonicotinoids, pyrazoles, and oxadiazines [10]–[12]. Consequently, it is urgently needed to create novel insecticidal agents to overcome the above problems.

RNA interference (RNAi) is an evolutionarily conserved genetic regulation mechanism that permits to down regulate gene expression in many eukaryotes, including insects. It causes sequence-specific mRNA degradation by 21–23 nucleotides and small interfering RNAs (siRNA) generated from longer dsRNAs by ribonuclease III cleavage activity [13]–[15]. This technique has been recently used and developed into new biological pesticides, although improvements are needed. Kumar et al. (2009) [16] found that silencing an acetylcholinesterase gene of *Helicoverpa armigera* (Hübner) (Lepidoptera: Noctuidae) by chemically synthesised siRNA led to larval mortality and growth inhibition, and Gong et al. (2011) [17] reported that knockdown of Rieske iron–sulfur protein gene expression by chemically synthesized siRNA at a concentration of 3.0 µg cm^−2^ induced 73% death of *P. xylostella*, suggesting the future use of chemically synthesized and modified siRNA as a novel bio-pesticide in the near future.

In the present study, we attempt to test chemically synthesized siRNA as oral insecticide not only in the laboratory, but also under field conditions. The siRNA we tested was designed to target *P. xylostella* AChE1 and AChE2 genes. We monitored mortality caused by the siRNA and also checked the level of down-regulation of the transcripts as well as the AChE activity. Effects of the siRNA on treated *Brassica oleracea* and *B. alboglabra* plants were also investigated with the different dosages used. Based on our data we discuss creating novel bio-pesticides based on the RNAi technology by chemically synthesizing siRNAs.

## Materials and Methods

### Ethics Statement

No specific permissions were required for these locations or activities. The location is not privately-owned or protected in any way. The field studies did not involve endangered or protected species.

### Insect Rearing

A population of *P. xylostella* was originally collected from insecticide-free cabbage field, which was maintained on the cabbage leaves in standard conditions set at 25±1°C, 16: 8 h light: dark photoperiod and 60–70% relative humidity until pupation.

### Small Interfering RNA and Formulation

The special siRNAs (Table 1) were designed to target the cDNA sequence of *P. xylostella* AChE genes (GenBank numbers: AY061975 and AY970293). The siRNAs were chemically synthesized by Guangzhou RiboBio Co Ltd, and were modified by addition of a dTdT overhang in the 3′end, 2′-methyl-nucleotides, and 5′ polyethylene glycol (PEG). The formulation is a sodium salt containing chitosan and 18–27 base pairs of double-stranded oligonucleotide at the concentration of 0.006%–0.01%. Its chemical formula is C_400–600_H_500–500_N_50–260_Na_10–60_O_250–420_P_40–60_ and the content is more than 75%. Physical properties of this product are: crystal powder with the color of white to slight yellow, odorless and easy to deliquescence, soluble in water and dimethyl sulfoxide.

**Table 1:** Chemically synthesized and modified siRNA from *P. xylostella* AChE1 (Si-ace1_001, Si-ace1_002 and Si-ace1_003) and AChE2 (Si-ace2_001, Si-ace2_002 and Si-ace2_003).

Si-ace1_001	5′-CATGCATGGTGATGAAATA-3′
Sense	5′-CAUGCAUGGUGAUGAAAUATT-3′
Anti-sense	3′-TTGUACGUACCACUACUUUAU-5′
Si-ace1_002	5′-GAATGATGTTGCCAGACAA-3′
Sense	5′-GAAUGAUGUUGCCAGACAATT-3′
Anti-sense	3′-TTCUUACUACAACGGUCUGUU-5′
Si-ace1_003	5′-CAGAGAGGAGAGTGTGATA-3′
Sense	5′-CAGAGAGGAGAGUGUGAUATT-3′
Anti-sense	3′-TTGUCUCUCCUCUCACACUAU-5′
Si-ace2_001	5′-CGGCGACACTTGATCTATA-3′
Sense	5′-CGGCGACACUUGAUCUAUATT-3′
Anti-sense	3′-TTGCCGCUGUGAACUAGAUAU-5′
Si-ace2_002	5′-CAGACACGATGATGAAAGA-3′
Sense	5′-CAGACACGAUGAUGAAAGATT-3′
Anti-sense	3′-TTGUCUGUGCUACUACUUUCU-5′
Si-ace2_003	5′-CTGGCTATTCGTTGGATAA-3′
Sense	5′-CUGGCUAUUCGUUGGAUAATT-3′
Anti-sense	3′-TTGACCGAUAAGCAACCUAUU-5′

### Laboratory Bioassay

The experiment was designed with two round tests. i: the aim of the first bioassay was to confirm which siRNA had the best effectiveness to kill the insect. The method was the same as described previously [17]. Briefly, each siRNA was uniformly coated on one side of cabbage leaves to form sandwiches at a concentration of 3 µg cm^−2^. Thirty two second-instar larvae of *P. xylostella* were used for each siRNA treatment and the insects were starved for 12 h before testing. The negative control was performed using DEPC water. The larvae were allowed to feed on the treated leaves for 72 h at 26±1°C, 60–70% RH and 16: 8 h (light:dark) photoperiod, and the larval mortality was recorded after 72 h. The larvae were considered to be dead when they stopped moving in response to touch. ii: according to the mortality detected in the above testing, we selected the most efficient siRNA (Si-ace2_001) to use in subsequent experiments. The toxicity of Si-ace2_001 against the second-instar larvae of *P. xylostella* was investigated *in vitro* by using a leaf-spray method, which is a standard routine test accepted by the IOBC/WRPS Working Group on “Pesticides and Beneficial Arthropods” [18]. The Si-ace2_001 was dissolved and diluted in DEPC water to obtain concentrations of 0 (control), 12.5, 25, 50, 100, 200 (µg/ml). Fresh cabbage discs (2 cm diameter) were placed on wet filter paper in a Petri dish (9 cm diameter). Ten second instar larvae of *P. xylostella* were transferred to leaf and sprayed (1 mL solution) by potter spray tower (Auto-Load; Burcard® Scientific), each concentration of Si-ace2_001 was applied with four replicates and a DEPC water control. The treated insects were maintained in the standard conditions described above until mortality was recorded after 72 h.

### Quantitative Real-time PCR

Ten insect samples were prepared from the laboratory bioassay for total RNA extraction. RNA samples were treated with DNase I (TakaRa, Japan) to remove any genomic DNA contamination. The first-strand cDNA was synthesized from total RNA (600 ng) with SuperScript III (Invitrogen, UAS), and the cDNA was subsequently used as PCR template for amplification, and detection of the specific *PxAce2* sequence. The relative mRNA expression of *PxAce2* was assessed by qPCR using primers as following: (Forward) 5′ CAGGAGAGAGGGCACAGGACATTG 3′ and (Reverse) 5′ TAGGCAGAAATACCCCATCAACCGT 3′. The qPCR reaction was performed in a total volume of 20 µl with 10 ng of cDNA, 0.2 µM primers and SYBR® Premix Ex Taq™ (TaKaRa) in the BioRad iQ5 Real-Time PCR Detection System. The qPCR was repeated with three technical replicates for each of three independent biological replicates (independently-extracted total RNA). The PCR Cycling conditions were composed of 95°C for 3 min, followed by 40 cycles of 95°C for 10 s, 58°C for 10 s, 72°C for 30 s. A dissociation step cycle at 95°C for 10 s and 60°C for 15 s, for melting curve analysis, was added as a final step. After qPCR, the homogeneity of PCR product was confirmed by melting curve analysis. Expression of *actin* gene was used as an internal standard to normalize the amount of other transcripts with the primers as following: (forward) 5′-CTGGACCTGCCTCATCATAC-3′and (reverse) 5′-GTGCTCAGTGGTGGAACAAC-3′ [19]. Relative transcript level of *PxAce2* was first normalized to the endogenous reference gene *actin* by the following equation: ratio = 2^−ΔΔCt^ [20], and then normalized relative to the level of gene transcripts in control insects.

### Determination of *PxAce2* Activity

Approximately, 3 g of whole body larvae were homogenized in 30 ml of ice-cold phosphate buffer (pH 7.4) containing 1∶10 (w/v) with 1 mM EDTA and 0.5% (V/V) Triton X-100. The homogenates were centrifuged at 13,000g for 20 min at 4°C, and AChE activity was immediately measured in the supernatant at 25°C according to Ellman [21], [22]. Briefly, the supernatant were assayed for activity with ATC (0.5 mM) as a substrate, enzymatic activity was recorded at 410 nm for 5 min in a Benchmark microplate reader (Bio-Rad). The enzymatic activity was corrected by calculating of AChE activity as in units of µmol ATC hydrolysed/min/mg.

### Field Experiment

The field experiment was done in a cabbage farm located at Baiyun district (Latitude: 23.158015 Longitude: 113.273070) Guangzhou, China, which was arranged in a randomized complete block design with the following six treatments: 0 (control), 12.5, 25, 50, 100, 200 (µg/ml). Each treatment was repeated three times with each one in a plot (15 m^2^). *P. xylostella* populations were assessed prior to insecticide application by counting the number of *P. xylostella* on 5 individual plants per plot. The Si-ace2_001 was mixed with 0.1% Tween-20 for application with an electric sprayer (China), and the mortality was recorded at 1, 3, 5 and 7 days. Decamethrin (2.5% EC) was used as a positive control and the percentage of mortality rate was corrected through Abbott’s formula [23].

### Safety Study of siRNA

Two Cruciferae vegetables (*Brassica oleracea* and *Brassica alboglabra*) were treated by chemically synthesised Si-ace2_001 to evaluate its safety towards treated plants. *B. oleracea* and *B. alboglabra* were planted in the vitreous greenhouse of South China Agricultural University (Guangzhou, China) under the temperature of 23±2°C. The plants were treated with Si-ace2_001 in the vegetative growth period to evaluate growth rate of stem, plant morphology, and damage to leaf color. The plants were ensured to be free of any other pesticides. *B. oleracea* and *B. alboglabra* were sprayed with Si-ace2_001 with the concentrations of 50, 100 and 200 µg mL^−1^, and controls (untreated). Experimental crops, planted in diameter 20 cm and high 15 cm tile basin, were divided into four groups with four replicates in each group and each replicate with four plants. Each plant was sprayed completely until the dripping of reagent from the leaves. Plant height was measured before the treatment and measured again at 3 and 9 days after treatment. Growth rate of stem were calculated by R = L/D, in which R is growth rate (cm/d); L is plant growth promotion in the experimental plants (cm); D is experimental time interval (days).

### Statistical Analysis

All data are represented by the mean±SEM. The difference between Si-ace2_001 treated and un-treated groups were separated based on *t-*test with p<0.05 representing significance using Sigma Plot 12.0 software (Systat Software Inc.).

## Results

### Toxicity of Si-ace2_001 against *P. xylostella* from Laboratory and Field Bioassays

**Figure 1:**
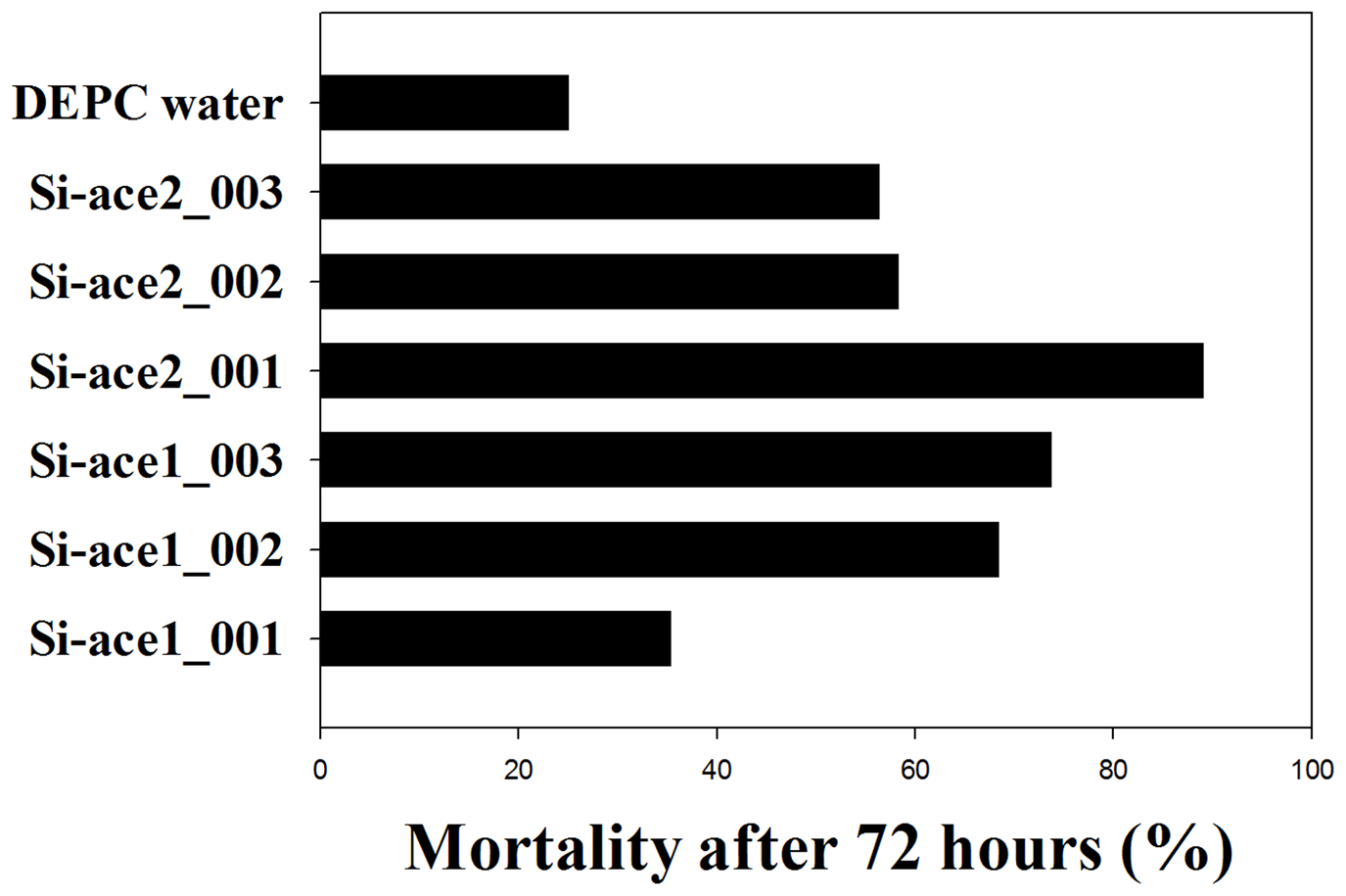
Mortality caused by chemically synthesized siRNA against second-instar larvae of *Plutella xylostella,* assessed by feeding test after 72 hour.

**Figure 2:**
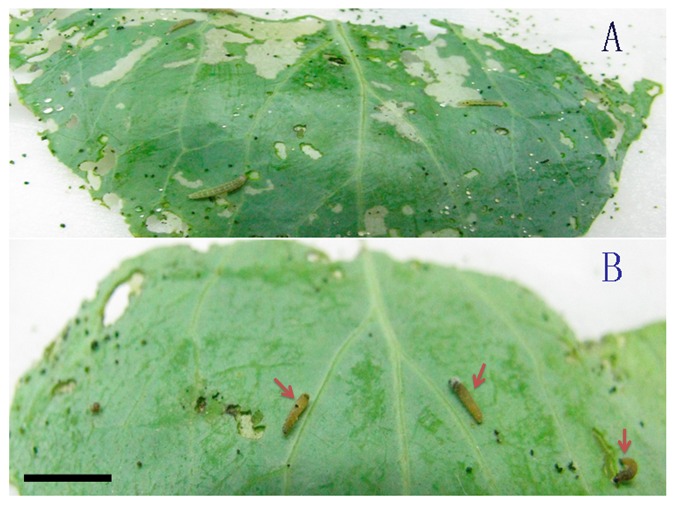
Laboratory bioassay of Si-ace2_001 against the larvae of *Plutella xylostella.* A is a negative control treated by DEPC water. B showed the samples treated with Si-ace2_001 and red arrowheads indicated the dead insects. Scale represents 1 cm.

**Figure 3:**
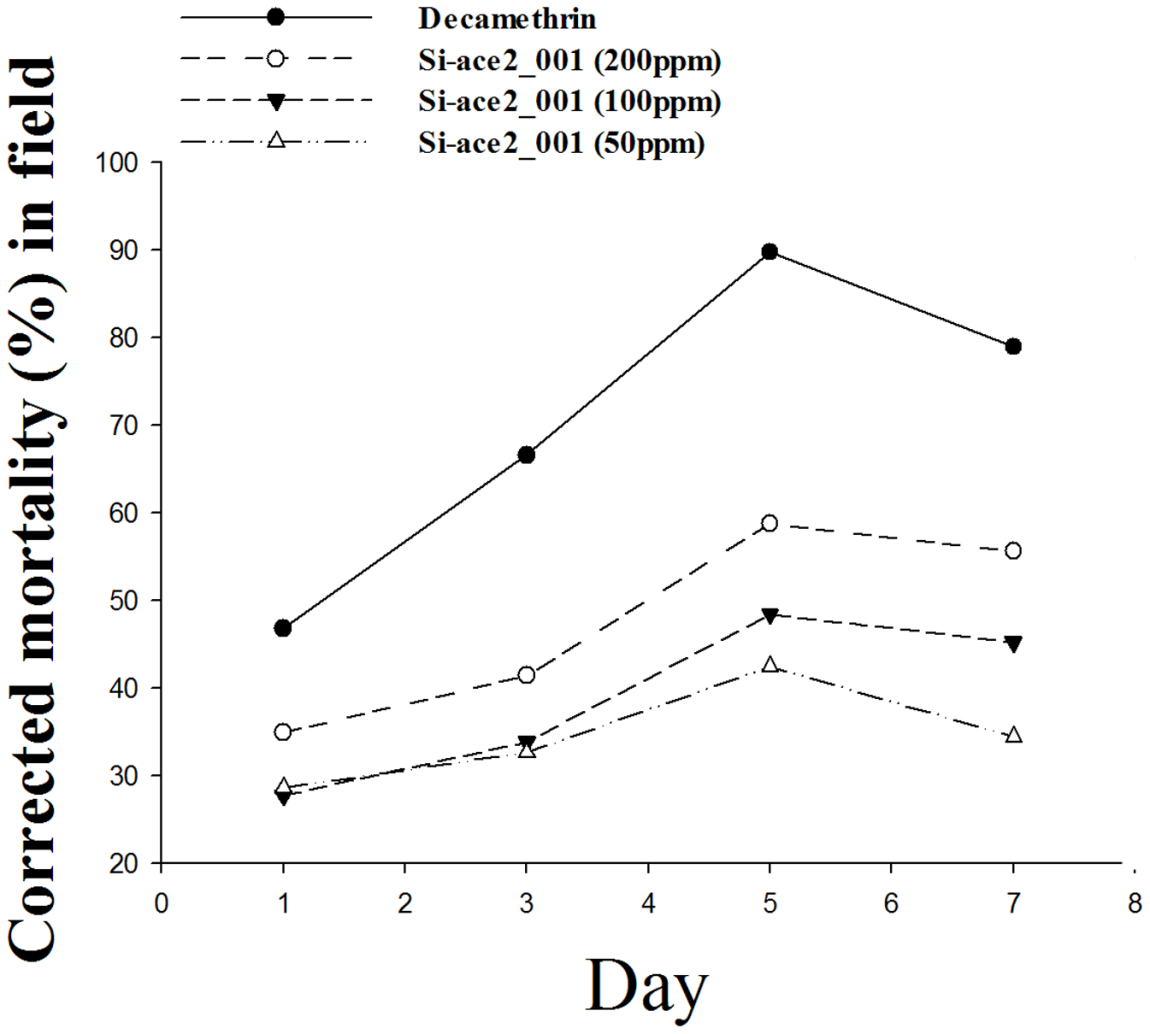
Corrected mortality of Si-ace2_001 against the larvae of *Plutella xylostella* in the field trial. Decamethrin (2.5% EC) was used as a positive control.

Two *P. xylostella* AChE genes were targeted by chemically synthesized siRNAs. We detected that Si-ace2_001which had the highest insecticidal effectiveness, causing 89% mortality at 72 h after exposure (Figure 1). In the case of *P. xylostella* AChE1, Si-ace1_003 was shown the most insecticidal against *P. xylostella*, inducing 73.7% mortality (Figure 1). The acute lethal concentrations of LC50 and LC90 for Si-ace2_001 were 53.66 µg/ml and 759.71 µg/ml, respectively (Table 2, Figure 2). Lethal concentration LC50 of Si-ace2_001 with corresponding 95% fiducial limits of the upper confidence limit and the lower confidence limit were 72.44 µg/ml and 39.76 µg/ml, respectively (Table 2). Treatment with the Si-ace2_001 in field bioassays for 5 days at concentrations of 200 µg/ml, 100 µg/ml and 50 µg/ml resulted in 58.8%, 48.4% and 42.4% mortality, respectively. This mortality was even higher than the 7 days after treatment (Figure 3, Table 3). The positive control of Decamethrin (2.5% EC) showed a strong toxicity towards *P. xylostella* with 89.8% at 5 days and 79.0% at 7 days (Figure 3, Table3).

**Table 2:** Lethal concentration values (LC50 and LC90) of Si-ace2_001 to second stage larvae of *P. xylostella*, assessed in laboratory with oral toxic test.

		LC50 (Fiducial Limits)				
LC90(µg/µl)	LC50(µg/µl)	Lower(µg/µl)	upper(µg/µl)	R	Slope	Intercept
**759.71**	**53.66**	**39.76**	**72.44**	**0.98**	**2.53**	**1.43**

**Table 3:** Effects of Si-ace2_001 on the larvae of *P. xylostella* in the field trial.

		After 1 day		After 3 day		After 5 day		After 7 day	
Concentrations(µg/µl)	Total insectNo. beforetreatment	LivingNo	Reducingpercentage(%)	LivingNo	Reducingpercentage(%)	LivingNo	Reducingpercentage(%)	LivingNo	Reducingpercentage(%)
**200**	219	150	31.5	126	42.5	102	53.4	91	58.4
**100**	226	172	23. 9	147	35.0	132	41.6	116	48.7
**50**	210	158	24.8	139	33.8	137	34.8	116	44.8
**25**	295	228	22.7	175	40.7	198	32.9	181	38.6
**12.5**	194	169	12.9	152	21.6	160	17.5	141	27.3
**0**	282	297	−5.3	277	1.8	319	−13.1	264	6.4

Reducing percentage (%) was calculated by equation of (total insect number-living insect number)/total insect number×100.

### Alterations in Activity and mRNA Expression of *P. xylostella* AChE2

**Figure 4:**
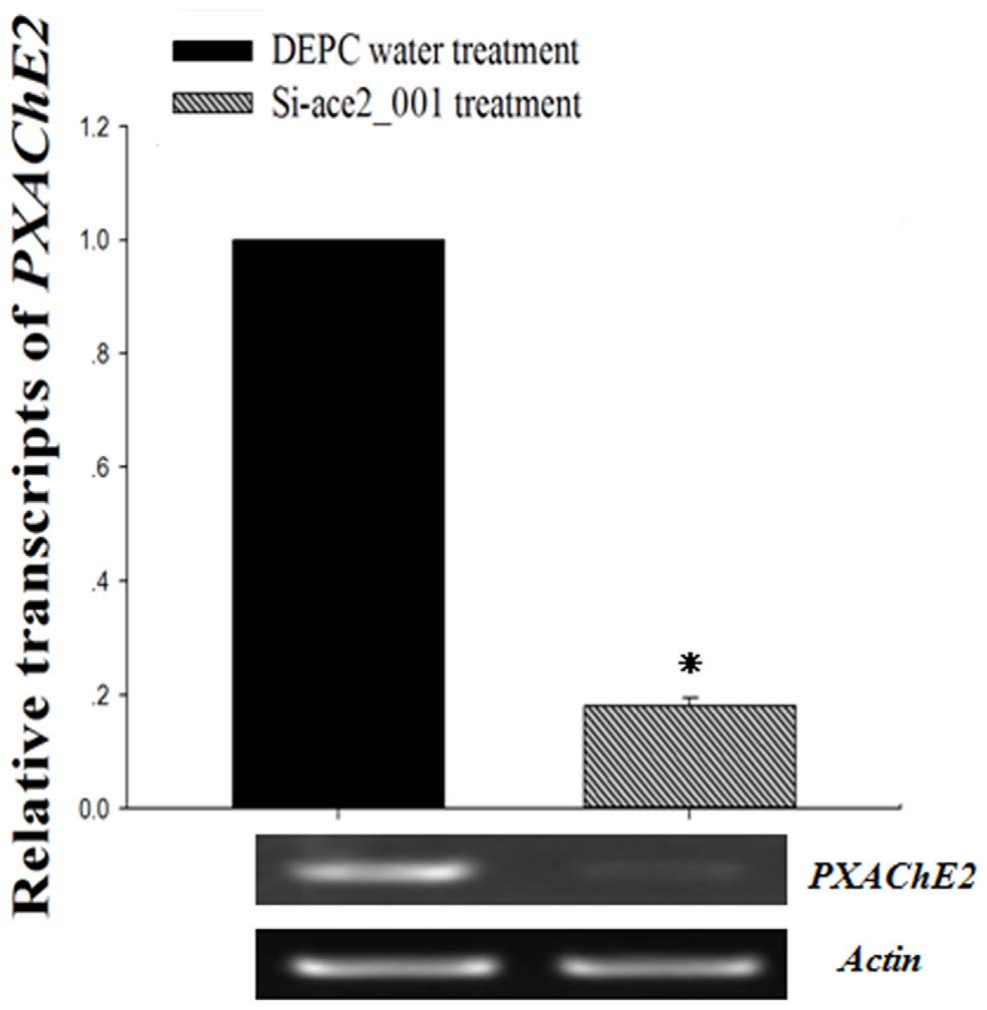
Real-time PCR data were normalized to the expression of *PxAchE2* in the samples treated with Si-ace2_001 or DEPC water as shown by RT-PCR gel pictures at the bottom of each panel. Each point represents the mean±SEM from three independent experiments and asterisk means significant differences (P<0.05) between the two groups.

**Figure 5:**
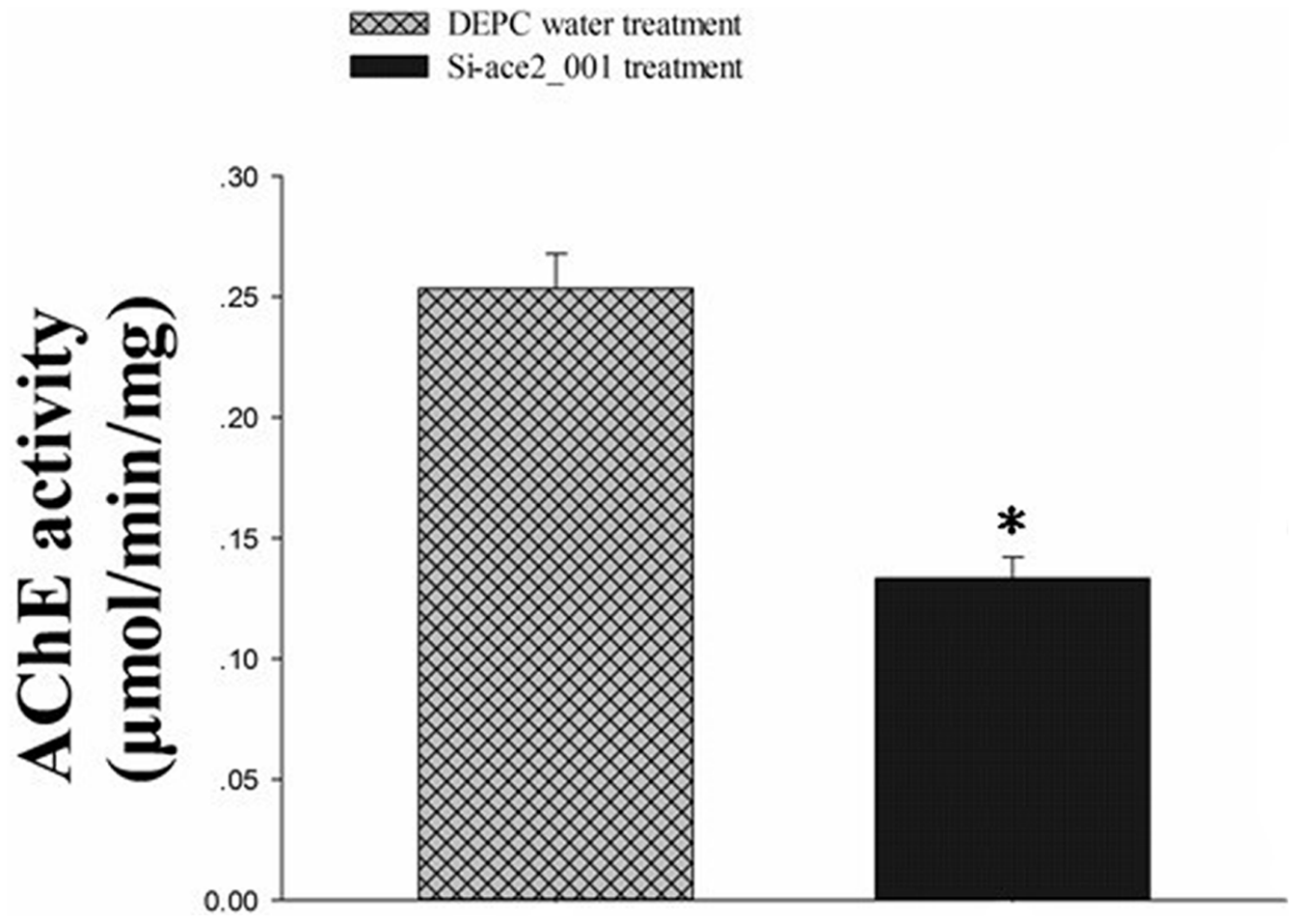
Activity of AChE from *Plutella xylostella* larvae treated by Si-ace2_001 or DEPC water. Each point represents the mean ±SEM from three independent experiments and asterisk means significant differences (P<0.05) between the two groups.

Effects of Si-ace2_001 on the mRNA levels of *P. xylostella* AChE2 are presented in Figure 4. Compared with the control group, a significant decrease (P<0.05) in mRNA levels of AChE gene was observed, with up to 5.7 fold down-regualtion. In the same treated samples, a significant decrease of AChE activity in the Si-ace2_001 treatment groups was observed with a 1.7 fold down-regulation (P<0.05) (Figure 5).

### Effects of Si-ace2_001 on Treated Plants

We did not detect any negative effects during treatment of planst with Si-ace2_001. In terms of plant morphology, color and growth of vein. The stem growth rate of *B. oleracea* was slightly increased by Si-ace2_001 treatment at the concentration of 200 µg mL^−1^ (P = 0.1) (Figure S1). No negative effects were showed on *B. alboglabra* plants treated with Si-ace2_001.

## Discussion

The most important finding of this study is the discovery that chemically synthesized and modified siRNA targeting *P. xylostella* AChE genes caused significant mortality of the insect both in the lab and field conditions, which provide a novel strategy to control *P. xylostella* and to develop bio-pesticides based on RNAi technology. The demonstration of RNAi in insects and its successful usage in disrupting the expression of genes opened a door to the development of a variety of novel, environmentally sound approaches for insect pest management [24]–[28]. dsRNA/siRNA can be delivered into insects through microinjection, oral feeding, soaking and transgenic expression [29]–[32], yet these methods can only be utilized under field conditions through the use of transgenic strategies, such as genetically modified plants and engineered bacterial strains expressing dsRNA [33], [34]. To the best of our knowledge, this is the first report demonstrating that siRNA prepared from chemically synthesis using modified nucleotides enhances stability and effectiveness of practical application of RNAi technology in field crops.

Extensive application of chemical pesticides have brought at least three drawbacks: significant increasing of insect resistance to pesticide; water, environment, and agricultural product contamination caused by residue of pesticide; and effects on non-target organisms result in upsetting the ecological balance [35], [36]. In addressing these issues, RNA is a natural and non-toxic material for non-targets, and chemically synthesized siRNA is able to effectively control the pest, suggesting that it may be defined as a new type of bio-pesticide. According to the LC50 values, toxicity of pesticides to *P. xylostella* were classified by IOBC category as <50% mortality is harmless or slightly harmful (N), 51–75% mortality is moderately harmful (M) and >75% mortality is harmful (T) [37]. Based on the field test data, Si-ace2_001 in the dose of 200 ppm has already reached to the level of moderately harmful (M), with mortality of 58.8% 5 days after exposure, although Si-ace2_001 showed lower mortality compared to 2.5% Decamethrin EC (corrected mortality is 89.8%) (Fig. 3).

Our targeted gene (AChE) is of vital importance in larval growth and development as investigated by RNAi-mediated knockdown of *AchE* transcript levels in *Helicoverpa armigera* [16], *B. germanica* [38] and *Chilo suppressalis* [39], indicating that insect AChE is an excellent target for pest control by using RNAi technology. In a similar study in *Tribolium castaneum*, silencing of *AchE* in 20-day old larvae by injecting 400 ng dsRNA led to >90% *AchE* transcript suppression, and approximately 2.5-fold AChE activity reduction, which caused 100% mortality within two weeks [40]. However, only 5.7-fold down-regulation of *AchE* transcripts and 1.7-fold reduction of AChE activity were achieved in our experimental conditions (Fig. 4 and Fig. 5). This discrepancy may be due to *T. castaneum* displaying higher susceptibility to systematic RNAi response compare to *P. xylostella* [41], [42]. It is generally accepted that AChE1 plays more significant roles in insect physiology compared with AChE2. Consequently, most reported mutations associated with resistance mainly occurred in *AChE1* in tested insects [43]–[45]. However, the mechanism underlying specific amino acid substitutions of AChE1 and AChE2 is not fully understood. To date, *AChE2* has also been reported to confer insecticide resistance in *Drosophila melanogaster* [46], *Lucilia cuprina* [47] and *Musca domestica* [48]. In our study, we found that silencing of *PxAChE2* caused higher mortality compared to *PxAChE1*, which may confirm the importance of *PxAChE2* in *P. xylostella*. Interestingly, the siRNAs targeting *PxAChE2* had different effects. For instance, the mortality of Si-ace2_002 and Si-ace2_003 were 58.3% and 56.3%, respectively, which is lower than the mortality caused by Si-ace1_002 (68.4%) and Si-ace1_003 (73.7%) (Fig. 1). Future studies should focus on determining an optimal target insect gene by using at least three different chemically synthesized siRNAs for insecticide activity screening.

Application of Si-ace2_001 with the formulation had no negative effects on plant morphology and growth. On the other hand, we detected evidence of functional acceleration of the growth of treated plants, possibly due to the formulation containing chitosan, which is primarily used for seed treatment and known as a plant growth enhancer [49], [50]. Chitosan also is known as a bio-pesticide substance, which can boost the innate ability of plants to defend themselves against fungal infections [51], [52]. The rationale for using chitosan in the formulation, was to enhance the efficacy of RNAi, as it was done before in insects [53] and mammals [54], [55]. There are a number of studies reporting the insecticidal activity of chitosan against *Spodoptera* spp. and aphids [56], [57]. Notably, the chitosan concentration reported to result in 44% mortality of *P. xylostella* larvae after 72 hours (similar to our bioassay conditions) was 1.2 g/L [58], which we estimate is more than 120-fold higher than the concentration used in our formulation (estimated at 10 µg/ml). Based on this difference we think the effect of chitosan to the *P. xylostella* larvae in our experimental conditions should be minimal.

**Figure 6:**
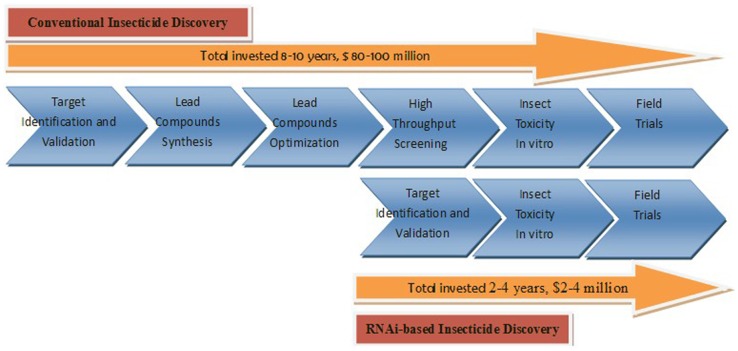
Schematic diagrams of comparing between conventional insecticide discovery and RNAi-based insecticide discovery.

Over the past 60 years, synthetic chemically pesticides have served as main tactic in the field of crop protection, but their availability is now declining as a result of the evolution of insect resistance [35], [59]. Therefore, alternative pest management agents are needed. RNAi has not only already provided a gold standard for validating the function of genes in basic research, but has also developed into a new pest control technique [29], [31], [33], [34], [60]. Current records indicated that the cost and time invested in bringing a new chemical to market have ballooned to $ 80–100 million and 8–10 years, respectively [61], [62]. To prevent further increases in the time and cost of developing new pesticides, chemically synthesis of siRNA may provide the fastest production capability, and the easiest scalability to insecticide activity screening. Calculating the time and cost involved in creating bio-pesticide from chemically synthesis siRNA is 2–4 years and $2–4 million, respectively (Fig. 6). In addition, RNAi-based insecticide discovery displays obvious advantages compared to conventional insecticide discovery. Future work will focus on improving stability, siRNA potency and testing potential non-target effects.

## Supporting Information

Figure S1 A: *Brassica oleracea* was treated with Si-ace2_001 at the concentration of 200 µg/ml after the exposure 9 days (Π), and un-treated samples (Ι). B: The growth rate of stem of *B. oleracea* treated by Si-ace2_001 at the concentration of 200 µg/ml after exposure 9 days. Error bars indicate SEMs from four replicates and each one consists of four individuals.
(TIF)
