# Supplementary figures and images for: Testing Insecticidal Activity of Novel Chemically Synthesized siRNA against Plutella xylostella under Laboratory and Field Conditions
Source: PLoS One. 2013 May 7;8(5):e62990. doi: 10.1371/journal.pone.0062990 (PMC3646892; doi:10.1371/journal.pone.0062990)

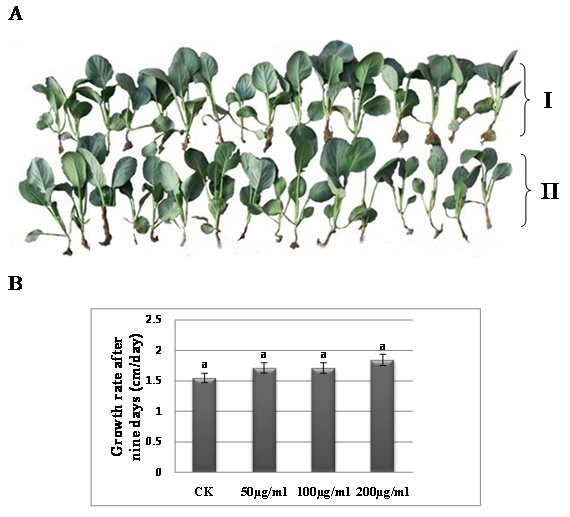

Supplement: Figure S1 — A: Brassica oleracea was treated with Si-ace2_001 at the concentration of 200 µg/ml after the exposure 9 days (Π), and un-treated samples (Ι). B: The growth rate of stem of B. oleracea treated by Si-ace2_001 at the concentration of 200 µg/ml after exposure 9 days. Error bars indicate SEMs from four replicates and each one consists of four individuals. (TIF) [file pone.0062990.s001.tif]
